# Supplementary material for: Systemic Barriers to Optimal Cancer Care in Resource-Limited Countries: Jordanian Healthcare as an Example
Source: Cancers (Basel). 2024 Mar 11;16(6):1117. doi: 10.3390/cancers16061117 (PMC10968872; doi:10.3390/cancers16061117)
Supplement: Supplementary file 1 [file cancers-16-01117-s001.zip › cancers-2900886-supplementary.pdf]

# Systemic Barriers to Optimal Cancer Care in Resource-Limited Countries: Jordanian Healthcare as an Example

Supplemental File A: Detailed Database Search Strategy

***PubMed***

(("Neoplasms/therapy"[Mesh] OR "Oncology Service, Hospital"[Mesh] OR "Health Services Accessibility"[Mesh] OR "Health Facilities"[Mesh] OR "Health Personnel"[Mesh] OR "Workforce"[Mesh] OR "Professional Practice"[Mesh] OR "Quality Assurance, Health Care"[Mesh] OR "Health Knowledge, Attitudes, Practice"[Mesh] OR "Health Literacy"[Mesh] OR "Health Education"[Mesh] OR "Health Promotion"[Mesh] OR "Health Policy"[Mesh] OR "Insurance, Health"[Mesh] OR "Insurance Coverage"[Mesh] OR "Healthcare Disparities"[Mesh] OR "Refugees"[Mesh] OR "Public Policy"[Mesh] OR "Health Expenditures"[Mesh] OR "Social Determinants of Health"[Mesh]) AND ("Jordan"[Mesh] OR "Jordanians"[Mesh]))

## *EMBASE*

('cancer care' OR 'oncology services' OR 'healthcare infrastructure' OR 'medical services' OR 'healthcare facilities' OR 'hospital infrastructure' OR 'healthcare accessibility' OR 'oncology professionals' OR 'healthcare workforce' OR 'healthcare personnel'  OR 'workforce capacity' OR 'cancer care quality' OR 'healthcare quality' OR 'quality management' OR 'quality indicators' OR 'cancer awareness' OR 'health literacy' OR 'public education programs' OR 'health campaigns' OR 'health education' OR 'community outreach' OR 'health communication' OR 'health insurance' OR 'health coverage' OR 'health disparities' OR 'socioeconomic status' OR 'health equity' OR 'access barriers' OR 'financial barriers' OR 'insurance coverage' OR 'refugee healthcare' OR 'refugee cancer care' OR 'refugee health services' OR 'healthcare access' OR 'healthcare challenges' OR 'healthcare needs' OR 'vulnerable populations' OR 'government initiatives' OR 'financial assistance programs' OR 'healthcare policies' OR 'healthcare funding' OR 'healthcare subsidies' OR 'government support' OR 'policy interventions' OR 'cancer care initiatives' OR 'barriers to cancer care' OR 'healthcare interventions' OR 'healthcare strategies' OR 'healthcare solutions' OR 'policy recommendations' OR 'intervention outcomes' OR 'Jordanian healthcare system' OR 'healthcare outcomes' OR 'healthcare evaluation' OR 'healthcare assessment' OR 'healthcare impact' OR 'systematic review' OR 'narrative review' OR 'literature review methodology' OR 'review article' OR 'research synthesis' OR 'literature synthesis' OR 'narrative synthesis' OR 'review methodology') AND ('Jordan' OR 'Jordanian' OR ‘Amman’)

## *Web of Science*

(TS=("cancer care" OR "oncology services" OR "healthcare infrastructure" OR "medical services" OR "healthcare facilities" OR "oncology professionals" OR "healthcare workforce" OR "healthcare quality" OR "quality management" OR "cancer awareness" OR "health literacy" OR "public education programs" OR "health insurance" OR "health disparities" OR "socioeconomic status" OR "refugee healthcare" OR "government initiatives" OR "financial assistance programs" OR "healthcare policies" OR "barriers to cancer care" OR "Jordanian healthcare system" OR "systematic review" OR "narrative review")) AND (WC=("Jordan" OR "Jordanian"))

## Scopus

TITLE-ABS-KEY("cancer care" OR "oncology services" OR "healthcare infrastructure" OR "medical services" OR "healthcare facilities" OR "oncology professionals" OR "healthcare workforce" OR "healthcare quality" OR "quality management" OR "cancer awareness" OR "health literacy" OR "public education programs" OR "health insurance" OR "health disparities" OR "socioeconomic status" OR "refugee healthcare" OR "government initiatives" OR "financial assistance programs" OR "healthcare policies" OR "barriers to cancer care" OR "Jordanian healthcare system" OR "systematic review" OR "narrative review") AND (AFFILCOUNTRY("Jordan"))
